# *Burkholderia pseudomallei* Biofilm Promotes Adhesion, Internalization and Stimulates Proinflammatory Cytokines in Human Epithelial A549 Cells

**DOI:** 10.1371/journal.pone.0160741

**Published:** 2016-08-16

**Authors:** Chanikarn Kunyanee, Watcharaporn Kamjumphol, Suwimol Taweechaisupapong, Sakawrat Kanthawong, Suwin Wongwajana, Surasak Wongratanacheewin, Chariya Hahnvajanawong, Sorujsiri Chareonsudjai

**Affiliations:** 1 Department of Microbiology, Faculty of Medicine, Khon Kaen University, Khon Kaen, Thailand; 2 Department of Oral Diagnosis, Faculty of Dentistry, Khon Kaen University, Khon Kaen, Thailand; 3 Melioidosis Research Center, Khon Kaen University, Khon Kaen, Thailand; 4 Biofilm Research Group, Khon Kaen University, Khon Kaen, Thailand; University of Toledo College of Medicine and Life Sciences, UNITED STATES

## Abstract

*Burkholderia pseudomallei* is a Gram-negative bacterium that causes melioidosis. Inhalational exposure leading to pulmonary melioidosis is the most common clinical manifestation with significant mortality. However, the role of *B*. *pseudomallei* biofilm phenotype during bacterial-host interaction remains unclear. We hypothesize that biofilm phenotype may play a role in such interactions. In this study, *B*. *pseudomallei* H777 (biofilm wild type), *B*. *pseudomallei* M10 (biofilm mutant) and *B*. *pseudomallei* C17 (biofilm-complemented) strains were used to assess the contribution of biofilm to adhesion to human lung epithelial cells (A549), intracellular interactions, apoptosis/necrosis and impact on proinflammatory responses. Confocal laser scanning microscopy demonstrated that *B*. *pseudomallei* H777 and C17 produced biofilm, whereas M10 did not. To determine the role of biofilm in host interaction, we assessed the ability of each of the three strains to interact with the A549 cells at MOI 10. Strain H777 exhibited higher levels of attachment and invasion compared to strain M10 (*p* < 0.05). In addition, the biofilm-complemented strain, C17 exhibited restored bacterial invasion ability. Flow cytometry combined with a double-staining assay using annexin V and propidium iodide revealed significantly higher numbers of early apoptotic and late apoptotic A549 cells when these were infected with strain H777 (1.52%) and C17 (1.43%) compared to strain M10 (0.85%) (*p* < 0.05). Strains H777 and C17 were able to stimulate significant secretion of IL-6 and IL-8 compared with the biofilm mutant (*p* < 0.05). Together, these findings demonstrated the role of biofilm-associated phenotypes of *B*. *pseudomallei* in cellular pathogenesis of human lung epithelial cells with respect to initial attachment and invasion, apoptosis and proinflammatory responses.

## Introduction

Melioidosis, caused by the bacterium *Burkholderia pseudomallei*, predominantly affects people in regular contact with contaminated dust/soil or stagnant water in endemic areas e.g. northern Australia and north-east Thailand [1, 2]. Infection may occur by inhalation, ingestion or percutaneous inoculation. The global increase of melioidosis is of concern because of the high case-fatality rate, up to 61.5% [2–6]. Aerosol inhalation, particularly during heavy monsoonal rain and winds, appears to be the predominant form of transmission of *B*. *pseudomallei* and most cases have pulmonary involvement, leading to fulminant pneumonia and septic shock [1, 4, 7–9].

Formation of biofilm by *B*. *pseudomallei* during residence in host tissues and *in vitro* has been revealed [10–13] and has been associated with relapsing melioidosis in a cohort study of primary melioidosis patients in Thailand between 1986 and 2004 [14]. Moreover, biofilm formation by *Burkholderia* species, induced by lung environmental conditions, correlated with bacterial penetration and intracellular survival in host cells. This can lead to severe infections and frequent failures of antibiotic treatment in cystic fibrosis patients [15].

Numerous components of *B*. *pseudomallei*, including capsule [16] and pili [17], have been demonstrated to assist adhesion to host cells. After attachment to host cells, the pathogen enters cells using various factors i.e. the type 3 secretion system [18] and two-component response regulator (*irlRS*) [19]. Recently, *B*. *pseudomallei* SurE (stationary-phase survival protein E), located in a global transcriptional regulation operon, was demonstrated to play a role in the invasion of human lung epithelial cells [20]. Biofilm phenotypes in other species of bacteria are known to have enhanced ability to adhere to and interact with host cells. The biofilm polysaccharide of *Pseudomonas aeruginosa*, involved in bacterial surface adherence to A549 epithelial cells, facilitates flagellin-mediated NF-κB activation [21]. Biofilm-associated infections of *Staphylococcus epidermidis* exhibited not only greater cell attachment but also higher intracellular survival rates in monocyte-derived macrophages compared to their planktonic counterparts [22].

When airway epithelial cells encounter pathogens, innate defense and inflammatory responses are initiated. The host immune response to *B*. *pseudomallei* triggers TLR2, TLR4 and TLR5 receptors in epithelial cell lines and induces IL-8 production via NF-kB [23]. Murine respiratory epithelial cells produce major proinflammatory cytokines IL-6, TNF-α and chemokines via NF-κB and p38 MAPK pathways when challenged with *B*. *pseudomallei* [24]. In addition, Utaisincharoen and colleagues demonstrated that the interaction of *B*. *pseudomallei* with the human alveolar lung epithelium cell line A549 was sufficient to stimulate interleukin 8 (IL-8) production, a potent chemoattractant for polymorphonuclear cells [25]. Meanwhile, the biofilm phase *S*. *epidermidis* elicited lower amounts of pro-inflammatory cytokines, TNF-α and IFN-γ, but enhanced production of IL-8 from infected monocyte-derived macrophages, suggesting a silent course of biofilm-associated infections [22]. However, the roles of *B*. *pseudomallei* biofilm formation during bacterial molecular pathogenesis in human lung epithelial cells remain unclear.

Because melioidosis most frequently affects the lungs and the causative agent, *B*. *pseudomallei*, can produce biofilm, the goal of this study was to investigate the contribution of *B*. *pseudomallei* biofilm to bacterial pathogenesis at the cellular level and to stimulation of proinflammatory responses in human lung epithelial cells. Strains of *B*. *pseudomallei* used were H777 (a moderate biofilm producer), M10 (a biofilm mutant) [10] and C17 (a biofilm-complemented strain). Proinflammatory cytokines, including IL-6, IL-8, TNF-α and IFN-γ, were also assayed to gain a better understanding of *B*. *pseudomallei* biofilm involvement in innate immune responses.

## Materials and Methods

### Bacterial strains and cultures

Previously characterized *B*. *pseudomallei* strains used were H777 (the moderate biofilm-producing wild type) and M10 (the biofilm mutant) [10]. Strain C17, the biofilm-complemented strain, was constructed in this study as follows. *B*. *pseudomallei bpsl0618*, encoding a sugar transferase, was amplified from chromosomal DNA using the primers comp0618_for (5′-GGTACCGTGACACGACGGAACACGAAACGC**-**3′)/ comp0618 _rev (5′-CTCGAGTCAGAATGCGTTGCGGCCGACGAA**-**3′). Underlined sections of each primer indicate restriction sites, *Kpn*I and *Xho*I, used to clone the amplicon into pBBR1MCS [26] to generate pKCL0618. This plasmid was transformed into *Escherichia coli* S17-1 *λpir* and subsequently introduced into *B*. *pseudomallei* M10 by conjugation. The conjugants were selected on LB agar containing 64 μg/ml of gentamicin, 30 μg/ml of chloramphenicol and 50 μg/ml of tetracycline. The restoration of ability of the selected clone (named C17) to form biofilm was further determined by the quantitative microtiter-plate technique [10].

*B*. *pseudomallei* H777, M10 and C17 from frozen stocks were cultured on Luria-Bertani (LB) agar plate for 48 h at 37°C. A single colony of each strain was grown in 5 ml LB broth and incubated at 37°C with shaking at 200 r.p.m. for 18 h. The bacterial culture was adjusted to an optical density (OD) at 600 nm of 0.1 before further culture in LB broth at 2% inoculums (v/v) at 37°C with shaking at 200 r.p.m. to obtain mid-log phase or designated OD.

### Microscopic analysis of biofilm formation stained with FITC-ConA

Biofilms were allowed to form on a sterile glass slide within a sterile 50 ml conical tube containing 10 ml of OD_600_ 0.9 *B*. *pseudomallei* in LB broth and incubated at 37°C for 3 h to allow air-liquid interface biofilm formation. Thereafter, the glass slide with attached bacteria was gently transferred into a fresh medium and further incubated for another 24 h before being gently washed 3 times with sterile PBS. The biofilm formation step was repeated once to achieve the 2-day biofilm formation.

Biofilms were prepared for microscopic examination as previously described by Takenada and colleagues [27]. The biofilms were fixed with 2.5%v/v glutaraldehyde in PBS, pH 7.4 for 3 h at room temperature before 50 μg/ml FITC-ConA (Sigma) was added and incubated for 20 minutes, washed three times with sterile PBS and mounted with 80% v/v glycerol. For fluorescent microscopy, the FITC-ConA stained biofilms were visualized using a Nikon Eclipse Ni-U fluorescence microscope (20x Objective Plan Fluor; Nikon, Japan). FITC-ConA stained biofilms were also assessed using LSM-500 and LSM 800 confocal laser scanning microscopes (CLSM) (Zeiss, Germany). Image processing and data analysis was done using ZEN software.

### Cell lines

Cells of a human alveolar epithelial carcinoma cell line (A549) (CCL-185, American Type Culture Collection, MD, USA) were cultured in RPMI 1640 (Gibco BRL, Grand Island, NY) supplemented with 10% v/v heat-inactivated fetal bovine serum (FBS) (Gibco) (complete media) at 37°C in a humidified incubator under an atmosphere of 5% CO_2_ for all experiments.

### Adhesion assay

Approximately 1×10^6^ A549 cells were seeded into each well of a 6-well tissue-culture plate and incubated at 37°C overnight. The A549 cells were infected with mid-log phase *B*. *pseudomallei* (the pre-biofilm stage) at a multiplicity of infection (MOI) of 10 for 1 h at 37°C in 5% CO_2_ atmosphere. Nonadherent bacteria were removed by five gentle washes using sterile PBS. The A549 cells were then lysed with 0.1% v/v Triton X-100 to liberate the bacteria from the A549 cells and considered as adherent bacteria followed by serial dilution with sterile PBS and a drop plate technique on LB agar. The numbers of adhered bacteria were expressed as colony-forming units (CFUs) after incubation of the plates at 37°C for 48 h [28].

For microscopic observation, a sterile coverslip was placed in a 6-well plate then the A549 cells were infected as described above. After washes with PBS, cells were fixed with methanol for 1 min, stained with Giemsa for 3 min, washed with washing buffer for 5 min, rinsed with tap water and air-dried at room temperature. The coverslip was then mounted onto a glass slide and inspected under 100× oil-immersion objective magnification using a light microscope. The experiments were performed in triplicate on each of two independent occasions.

### Internalization and intracellular survival assays

Internalization and intracellular survival assays were performed as previously described by Techawiwattanaboon et al (2015) [20]. In brief, the A549 cells were infected with *B*. *pseudomallei* at MOI 10 for 2 h to allow bacterial internalization. The monolayers were then washed three times with sterile PBS before any remaining extracellular bacteria were killed using complete media containing 250 μg/ml kanamycin for another 2 h. For the intracellular survival assay, *B*. *pseudomallei* in A549 were monitored further at 8 and 12 h post infection (p.i.) in complete media containing 20 μg/ml kanamycin. After washing steps, the monolayers were lysed with 0.1% v/v Triton X-100. Serial dilutions of the lysate were prepared and bacteria present enumerated using a drop plate technique on LB agar.

Percentages of adhesion and of intracellular bacteria following internalization were calculated from three independent experiments.

### Cell cytotoxicity assay

The effect of *B*. *pseudomallei* biofilm on membrane permeability of infected A549 was determined according to the release of lactate dehydrogenase (LDH). Briefly, A549 cells were seeded into 6-well tissue culture plate at 37°C, overnight and infected with *B*. *pseudomallei* H777 and M10 at MOI 10 and 100 as described above, except that FBS was lowered to 5% to reduce background LDH activity. Culture supernatants (100 μl) were taken at 1, 4, 8, 12, 18 and 24 h p.i. and transferred into a new 96-well enzymatic assay plate for detection of LDH levels using the CytoTox96 kit (Roche) according to the manufacturer’s instructions. The reaction was measured using a microplate reader (Sunrise™, TECAN) at 492 nm. LDH levels of infected monolayers were normalized to maximum release of LDH (those corresponding to a 100% cell lysis, obtained by Triton X-100 lysis of a number of cells equal to that in infected samples). Cytotoxicity was calculated as follows:

$$\%\;cytotoxicity=\frac{experimental\;LDH-target\;spontaneous\;LDH}{maximal\;LDH-target\;spontaneous\;LDH}$$

where “experimental LDH” is the quantity of LDH released from infected, unlysed A549 cells; “maximal LDH” is the amount released from uninfected A549 cells after total lysis; and “target spontaneous LDH” is the amount released from unlysed, uninfected A549 cells.

Data were collected and analyzed from three independent experiments, each performed in triplicate.

### Analysis of A549 cells by flow cytometry for apoptosis and necrosis

Apoptosis and necrosis of A549 cells infected with *B*. *pseudomallei* H777 and M10 at MOI 10 at 37°C were determined at 12 h p.i. Apoptotic and necrotic cells were assessed according to the binding of annexin V and the uptake of propidium iodide (PI) (the Annexin V Apoptosis Detection Kit FITC (eBioscience)). Apoptotic cells were identified by flow cytometry on a FACS Calibur (Becton–Dickinson) equipped with a 488 nm argon laser light and a 623 nm band pass filter. The BD FACSDiva v.6.1.3 software (Becton-Dickinson) was used to process results. Logarithmic fluorescence intensity of annexin-V-FITC was plotted versus the fluorescence intensity of PI in a dot plot. A total of 30,000 epithelial cells were analyzed for each plot. The experiments were performed in triplicate on each of two independent occasions.

### Cytokine assays

The presence of IL-6, IL-8, TNF-α and IFN-γ in culture supernatants of A549 cells infected with *B*. *pseudomallei* H777, M10 and C17 strains at MOI 10 and 100 at 8 h p.i. were determined by ELISA (ELISA MAX Deluxe sets; Biolegend, San Diego, CA) according to the manufacturer’s instructions. Absorbance was measured at 450 nm using a microplate reader (Sunrise™, TECAN). Data were collected and analyzed from three independent experiments, each conducted with three replicates.

## Statistical analysis

Data are expressed as mean ± SD. Statistical analysis was performed using Statistics Package for the Social Sciences (SPSS) program version 16. Data were analyzed for statistical significance using the one-way ANOVA followed by Tukey’s honestly significant difference post hoc test. A statistically significant difference was considered at *p*-value of < 0.05.

## Results

### Microscopic analysis of biofilm phenotypes of *B*. *pseudomallei* H777, M10 and C17

**Fig 1:**
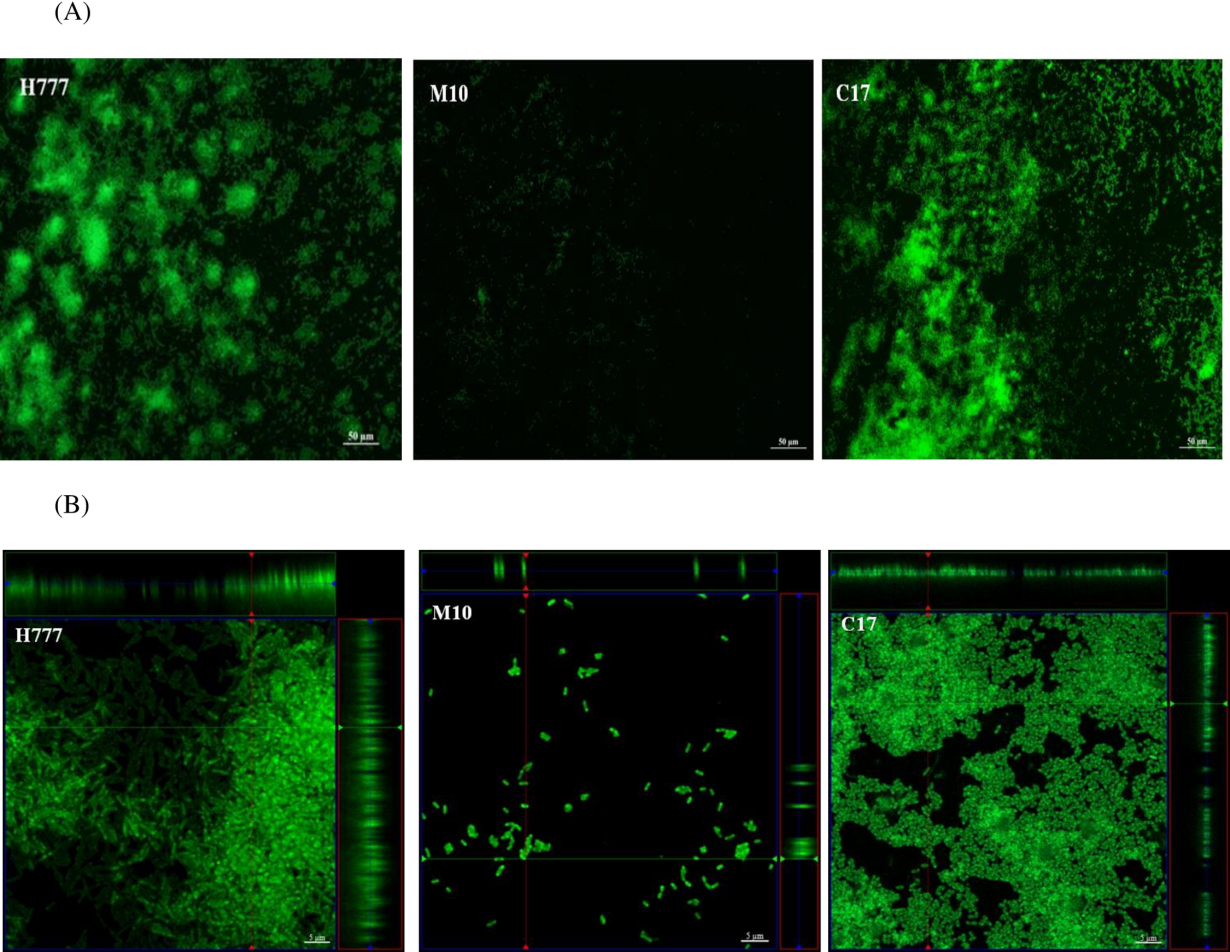
**(A)** Fluorescence microscopic views of the 2-day biofilms of *B*. *pseudomallei* H777, M10 and C17 grown statically on glass slides in LB broth at 37°C. The biofilms were stained with FITC-ConA and monitored under a Nikon Eclipse Ni-U fluorescence microscope (20× magnification). Strains H777 and C17 showed aggregation of surface-adherent bacteria whereas the biofilm mutant, M10, was rarely attached on the glass slide. (B) Confocal laser scanning micrographs of the 2-day biofilms of *B*. *pseudomallei* H777, M10 and C17 grown statically on glass slides in LB broth at 37°C. The biofilms were stained with FITC-ConA. The crossing lines in each images (x and y axes) indicate the correspondent vertical CLSM section (Z), indicating the thickness of the biofilm. The bars indicate 5 μm. The images were taken using a Zeiss 500 and a Zeiss 800 CLSM microscope (100× magnification).

Fluorescence images of the FITC-ConA-stained biofilm revealed the glass slide-adhered 2-day biofilms of *B*. *pseudomallei* (Fig 1A). Strain H777 (the biofilm wild type) and strain C17 (the biofilm-complemented strain) showed aggregation of surface-adherent bacteria. In contrast, the biofilm mutant, *B*. *pseudomallei* M10, was rarely attached to the glass slide.

CLSM of the 2-day biofilm of strain H777 stained with FITC-ConA demonstrated a thick layer of cells at the air-liquid interface (Fig 1B). The 2-day biofilm of strains H777 and C17 appeared to have covered the entire air-liquid interface area. However, structural differences were observed in the complemented strain, C17, compared to the wild type. In contrast, biofilm formation by the mutant strain M10 was impaired as only scattered cells of this strain had adhered to the glass slides (Fig 1B).

Collectively, these data indicate the ability of *B*. *pseudomallei* H777 and C17 to form biofilm in LB at 37°C whereas the ability of strain M10 to form biofilm was greatly impaired.

### Biofilm phenotype promotes *B*. *pseudomallei* adhesion to and internalization into A549 cells

**Fig 2:**
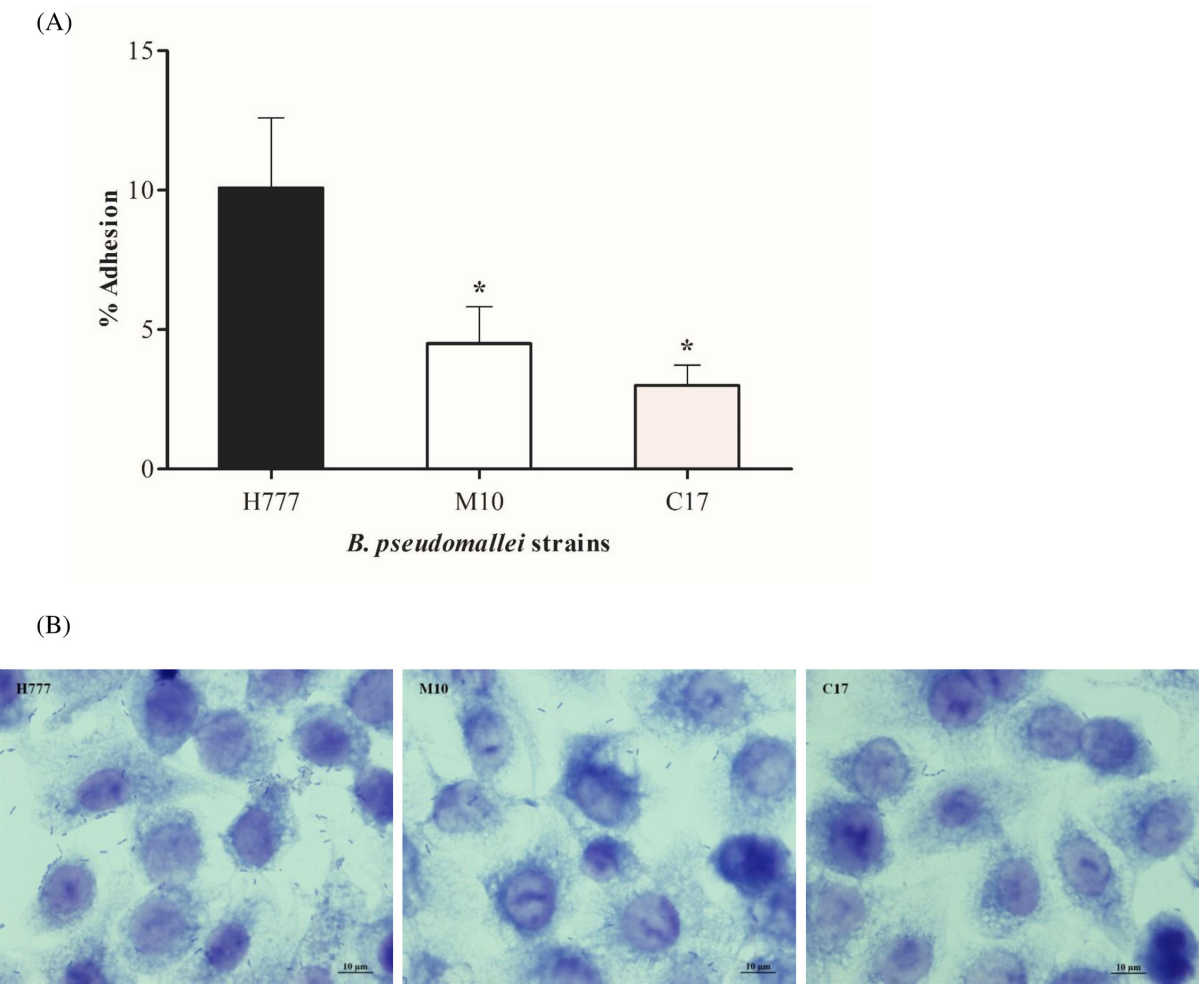
Adhesion of *B*. *pseudomallei* H777, M10 and C17 to human lung epithelial cells at MOI 10. (A) Percentages of bacterial adhesion were determined by comparing the number of adherent bacteria to the inoculum. The numbers of CFU of cell-associated bacteria were counted after 1 h p.i. using the drop plate technique. Data represent the mean ± standard deviation of triplicates from at least three independent experiments. Asterisks denote statistical significance relative to H777 (*p* < 0.05). (B) Light microscopic images demonstrating *B*. *pseudomallei* H777, M10 and C17 adhesion to A549 cells. Bar represents 10 μm. Representative images from Giemsa stained specimens and visualized under 100× magnification.

Adhesion of *B*. *pseudomallei* to airway epithelial cells followed by internalization are crucial for bacteria-host interactions. To address the question of whether these processes are associated with the biofilm phenotypes of *B*. *pseudomallei*, we performed adhesion and internalization experiments using strains with three different biofilm phenotypes. Numbers of cells adhering were significantly higher (*p* < 0.05) for strain H777 (wild type) (10.08±2.51%) relative to strain M10 (biofilm mutant) (4.50±1.32%). The complemented strain, *B*. *pseudomallei* C17, did not exhibit restored adhesion ability (2.99±0.73%) (Fig 2A). Giemsa staining observation under a light microscope confirmed that more cells of the biofilm phenotype, H777, adhered to the surface of A549 cells relative to the strains M10 and C17 (Fig 2B).

**Fig 3:**
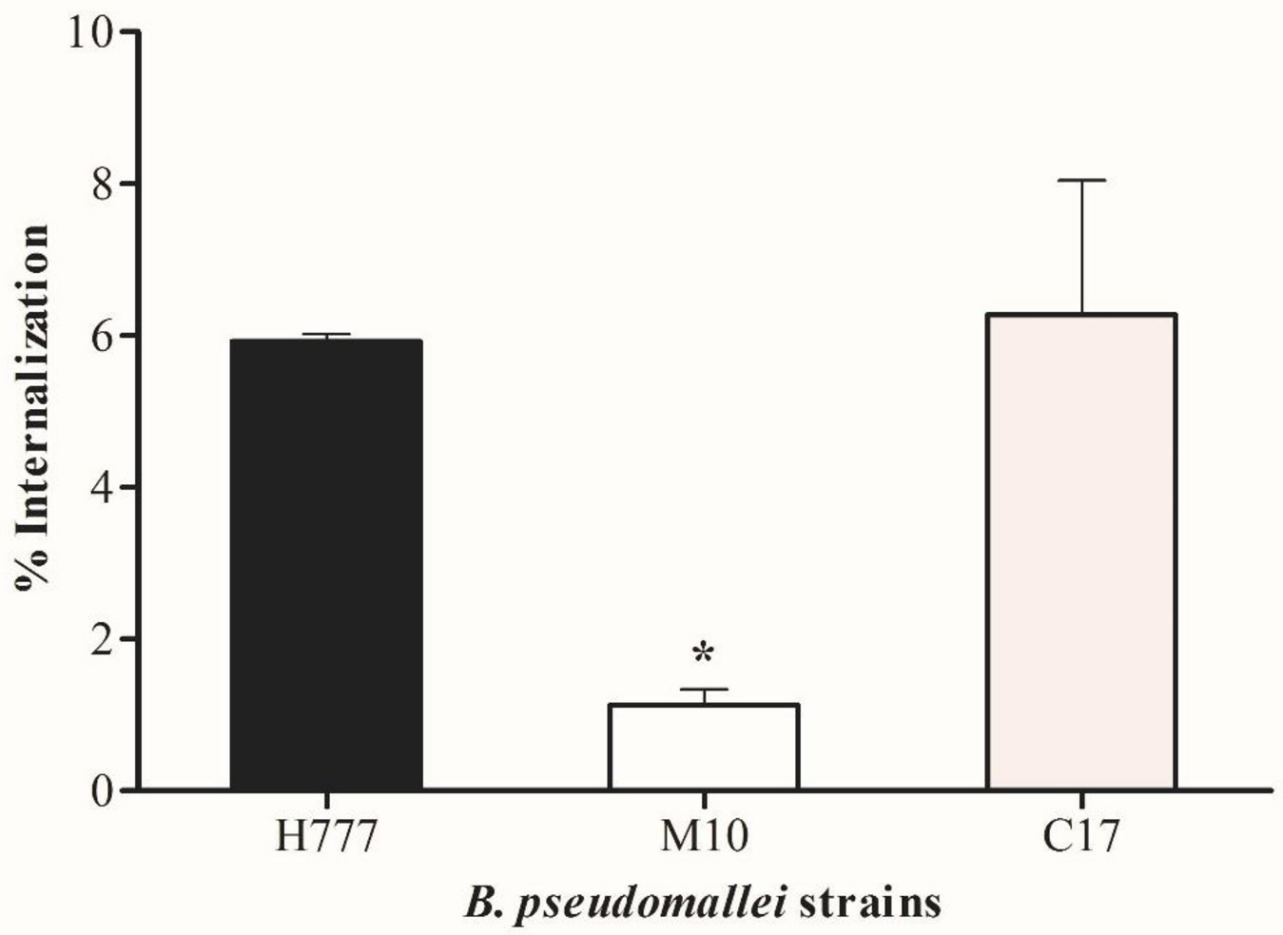
Internalization of *B*. *pseudomallei* H777, M10 and C17 strains into human lung epithelial cells at MOI 10. The number of bacteria internalized were enumerated at 4 h p.i. using the drop plate technique. Percentages of bacteria that successfully internalized were calculated from the number of intracellular bacteria compared to the inoculum. Data are represented as the means ± standard deviation from at least three independent experiments, each in triplicate wells. Asterisks denote statistical significance relative to H777 (*p* < 0.05).

Strain H777 correspondingly exhibited a significantly higher percentage of successful invasion into A549 cells (5.92±0.10%) than did the biofilm mutant, M10 (1.13±0.21%) (*p* < 0.05). The biofilm-complemented strain, C17, exhibited a restored invasion ability (6.27±1.77%) (Fig 3). Hence, the ability of *B*. *pseudomallei* to adhere to and internalize into airway epithelial cells was facilitated by the presence of biofilms. However, the inconsistency of adhesion and internalization abilities by strain C17, the biofilm complement were observed.

### Intracellular survival and multiplication of *B*. *pseudomallei* in A549 cells

**Fig 4:**
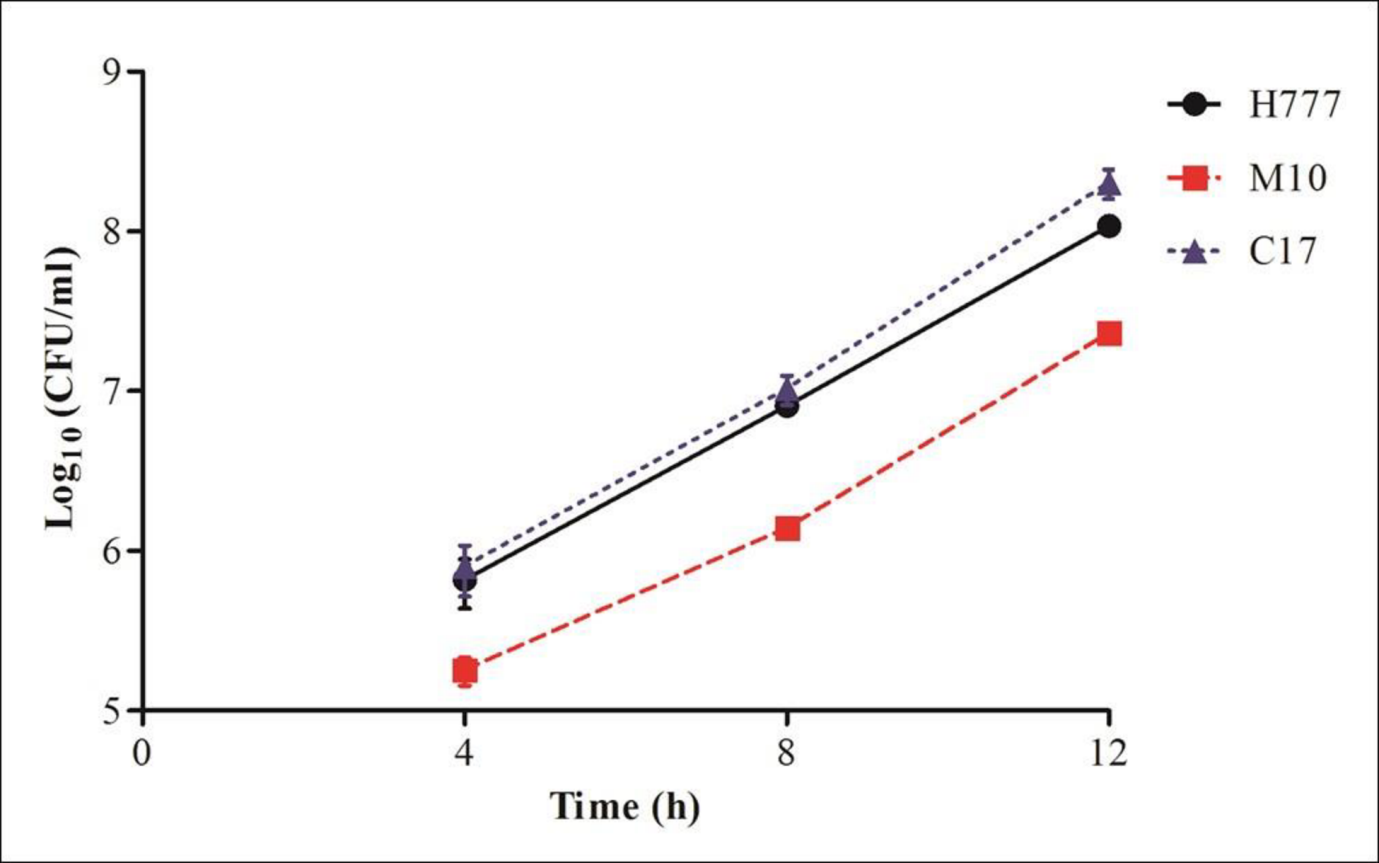
Intracellular survival and multiplication of *B*. *pseudomallei* H777, M10 and C17 strains in human lung epithelial cells at MOI 10. The number of bacteria present were enumerated at 4, 8 and 12 h p.i. using the drop plate technique. Data are represented as means ± standard deviation from at least three independent experiments in triplicate wells.

As an intracellular pathogen, *B*. *pseudomallei* requires virulence factors to facilitate intracellular survival and disease pathogenesis. Biofilm phenotypes of strains H777 and C17 were similar in their ability to internalize and multiply in the host cells (Fig 4) and were more successful in this than was the biofilm mutant, M10. The data imply that the biofilm phenotype can facilitate bacterial internalization but is not required for intracellular survival and multiplication in the human lung epithelial cells.

### Biofilm phenotypes contribute to apoptosis of *B*. *pseudomallei*-infected A549 cells

*B*. *pseudomallei* infection can result in cytotoxicity and apoptosis of infected cells [29–32]. One of the manifestations of cytotoxicity is the loss of cell membrane integrity with the consequent release of a cytosolic enzyme, LDH, into cell culture supernatants. The amounts of LDH released from human lung epithelial cells infected with any of the three strains used (H777, M10 or C17) at MOI 10 and MOI 100 increased in a time-dependent manner. MOI 100 produced a higher toxicity than did MOI 10 (data not show). However, the levels of released LDH from cells infected with either biofilm phenotype or the mutant were not significantly different. The findings revealed the potential damage or death induced in human lung epithelial cells infected by these three strains. However, the biofilm formation of the pathogen did not correspond to the human lung cell cytotoxicity.

**Fig 5:**
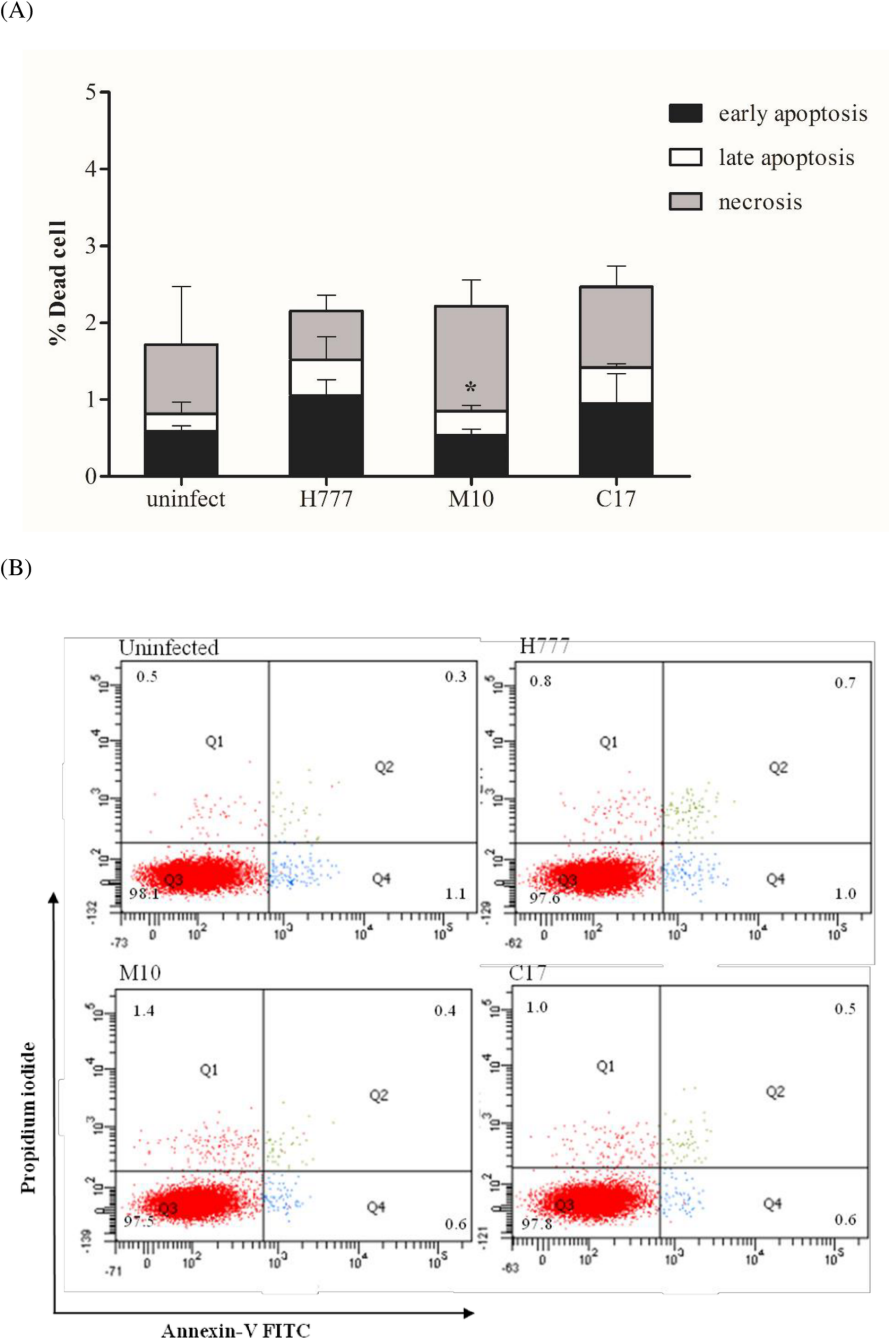
Induction of death among infected human lung epithelial cells with *B*. *pseudomallei* H777, M10 and C17 at MOI 10. At 12 h p.i. the apoptotic and necrotic cells were determined by flow cytometry. (A) Illustrates the percentage ± SD of dead A549 cells infected with *B*. *pseudomallei* H777, M10 and C17 strains. (B) Graphical representations of annexin-V/PI Plots. The numbers of viable (AV^-^/PI^-^), early apoptotic (AV^+^/PI^-^), late apoptotic (AV^+^/PI^+^) and necrotic (AV^-^/PI^+^) cells are shown. Asterisks denote statistical significance relative to H777 (*p* < 0.05).

The role of biofilm phenotype in apoptosis of infected lung epithelial cells was determined at 12 h p.i. using A549 cells infected with strains H777, M10 or C17 at MOI 10. Markers for apoptosis (annexin V, AV) or necrosis (propidium iodide; PI) were assayed using flow cytometry. The percentages of viable (AV^-^/PI^-^), early apoptotic (AV^+^/PI^-^), late apoptotic (AV^+^/PI^+^) and necrotic (AV^-^/PI^+^) A549 cells were determined. While focusing on total apoptotic cells (early apoptosis; black bar and late apoptosis; white bar), A549 cells infected with *B*. *pseudomallei* biofilm phenotypes, H777 and C17 exhibited significantly different proportions of early and late apoptotic cells (1.52 ±0.32% and 1.43 ± 0.40%) compared to M10 (0.85 ± 0.15%) (*p* < 0.05) (Fig 5A and 5B). In contrast, a higher proportion of cells were found to be necrotic (1.37±0.34%) when infected with the biofilm mutant, M10 while percentages of necrotic A549 cells infected with H777 and C17, the biofilm phenotypes, were 0.63±0.21% and 1.05±0.27%, respectively (Fig 5A and 5B).

The similar cell damage according to LDH released from the infected human lung epithelial cells by H777, M10 and C17 in parallel with the similar percentage of dead cells detected by flow cytometry. However, the biofilm phenotypes, H777 and C17 could induce more apoptosis suggesting the role of biofilm in this form of cell death during *B*. *pseudomallei* infection.

### Biofilm phenotypes influence cytokine production in *B*. *pseudomallei*-infected A549 cells

**Fig 6:**
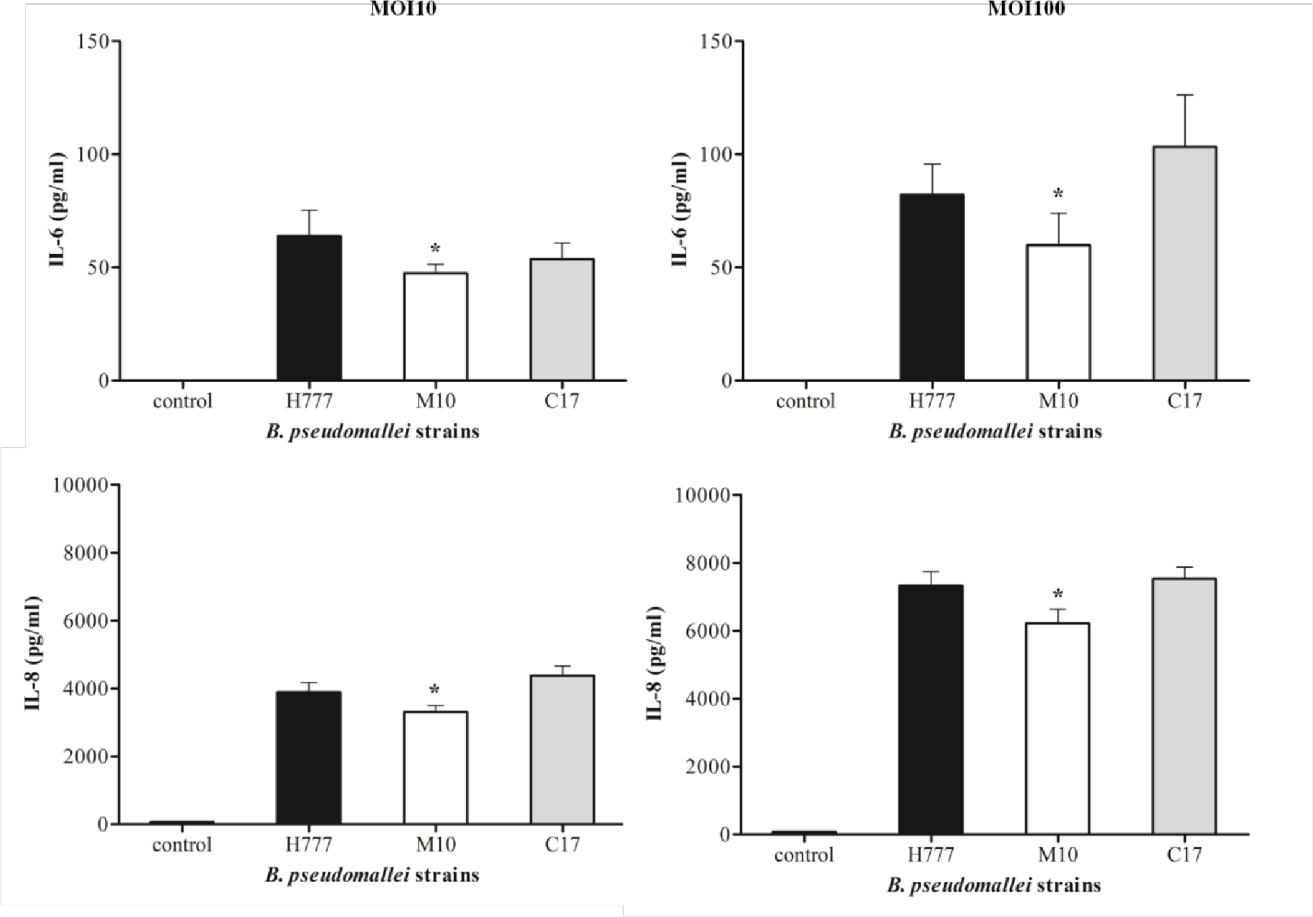
Cytokine production by human lung epithelial cells (A549) in response to infection with *B*. *pseudomallei* H777, M10 and C17 strains. A549 cells were infected at MOI 10 and MOI 100. The culture supernatants were harvested at 8 h p.i. to measure cytokine levels. Data represent the means ± standard errors for triplicate wells of single representative experiments. Each experiment was performed at least three times. Asterisks denote statistical significance (*p* < 0.05).

To further demonstrate the potential of biofilm phenotypes on inflammatory cytokine expression by *B*. *pseudomallei*-infected human lung epithelial cells, IL-6, IL-8, TNF-α and IFN-γ were assayed from supernatants harvested from the 8-h co-cultured experiments at MOI 10 and 100. A549 cells infected with strain H777 expressed a significantly higher level of IL-6 and IL-8 than did those infected with M10 (*p* < 0.05) at both MOI 10 and 100 (Fig 6). The levels of IL-6 and IL-8 at MOI 100 were 1.3–1.9 and 1.7–1.9 times higher than at MOI 10, respectively (Fig 6). Production of IL-6 at MOI 100 and IL-8 at MOI 10 and 100 in the complemented strain (C17) was restored. However, TNF-α and IFN-γ could not be detected from the A549 cells infected with any of the strains used (data not shown). These results indicate that biofilm phenotypes of *B*. *pseudomallei* stimulate A549 cell inflammation through increasing IL-6 and IL-8 production to a greater extent than does the non-biofilm producing strain.

## Discussion

Bacterial acquisition, attachment and invasion into the epithelial lining is a prerequisite of bacterial pathogenesis. Numerous reports have attempted to identify potent factors facilitating survival of *B*. *pseudomallei* in host cells and subsequent host responses such as apoptosis or necrosis of the infected cells. In this study, we demonstrated the biofilm phenotypes of *B*. *pseudomallei* strains H777 and C17 grown in LB at 37°C and that the biofilm mutant, strain M10, was deficient in this regard. The results are in agreement with those previously reported by Anutrakareunchai et al. [33] who demonstrated that *B*. *pseudomallei* could produce biofilm in both enriched and nutrient-limited conditions. In addition, the biofilm-forming ability of *B*. *pseudomallei* was also demonstrated in soil microcosms [34]. This confirms the ability of *B*. *pseudomallei* to form biofilm in a niche that can be either limited or enriched for nutrients.

A detailed knowledge at the molecular level of the interaction between *B*. *pseudomallei* and the eukaryotic cell surface is limited compared to that for other bacterial pathogens. Our study provides additional evidence for the importance of *B*. *pseudomallei* biofilm in bacterial attachment to the human lung epithelial cells since the biofilm phenotype H777 exhibited significantly higher adhesion to A549 cells than did the biofilm mutant, strain M10. The pre-biofilm stage of *B*. *pseudomallei* used in this study may be validated as the form of a biofilm live separately prior to the initial attachment to a surface [35]. This is in good agreement with the previous results by Lazar Adler et al [36]. The trimeric autotransporter adhesin, BbfA (*Burkholderia* biofilm factor A) was demonstrated to play role in biofilm formation, bacterial adhesion, microcolony formation of *B*. *pseudomallei* and virulence in a murine melioidosis model. Furthermore, *S*. *epidermidis*, the cause of hospital-acquired and biofilm-associated infections in the biofilm phase exhibited greater adhesion on monocyte-derived macrophages as compared to planktonic-phase bacteria, leading to a 10-fold higher intracellular survival in these macrophages [22]. *Pseudomonas aeruginosa* required biofilm-like properties to enter into airway epithelial cells, favoring bacterial survival and linked to antibiotic resistance and chronic infection [37]. Moreover, the polysaccharide Psl of *P*. *aeruginosa* was also reported to facilitate adhesion to A549 epithelial cells [21].

Rates of bacterial internalization and multiplication were significantly lower in the biofilm-defective mutant (M10) in A549 cells than in the wild type (*p* < 0.05): the ability to invade host cells was regained by the biofilm-complemented strain. These findings confirm the essential role of *B*. *pseudomallei* biofilm during bacteria-host cell interactions. This corresponds with the finding that biofilm-phase *S*. *epidermidis* ATCC35983 enjoys a 10-fold increase in internalization into macrophages relative to the planktonic phase. In addition, biofilm bacteria show greater intracellular survival [22]. Nevertheless, strain C17, the biofilm complemented strain demonstrated the weakest adhesion ability in contrast to the highest percentage internalization imply that biofilm formation may facilitate bacterial adherence and internalization to the host cells but may not be the sole requirement for these processes.

A number of virulence factors involved in cytotoxicity caused by *B*. *pseudomallei* infection have been reported. A *B*. *pseudomallei* mutant lacking a cell cycle-inhibiting factor (Cif) demonstrated a significant reduction in cytotoxicity and plaque formation in infected HeLa cells and U937 cells at 6 hours p.i. compared to the wild-type bacterial strain [38]. Cyclic diguanylic acid (c-di-GMP), an intracellular signaling molecule involved in motility, biofilm formation and virulence of *B*. *pseudomallei* was demonstrated to play a role in cytotoxicity on the infected human macrophage cells [32]. Moreover, phospholipase C enzyme 2 (Pcl-2) of *B*. *pseudomallei* was demonstrated to be toxic to the infected mouse macrophage [39]. These findings suggest that enzymatic disruption of the host cell membranes may contribute to *B*. *pseudomallei* virulence. This study demonstrated that the biofilm phenotype, *B*. *pseudomallei* H777, was more efficient at invasion and intracellular survival compared to the biofilm mutant M10. However, the cytotoxic effect of either biofilm wild type or the biofilm mutant on A549 cells was similar at all investigated times indicated the indistinguishable of the correspondence of *B*. *pseudomallei* biofilm to the death of infected human lung epithelial cells. However, the two general mode of cell death: apoptosis and necrosis appear to have different outcomes for the course of infection. We therefore further investigated whether the *B*. *pseudomallei* biofilm may manipulate the mode of death of infected host cells and may involve in microbial spread and enhance the induction of immunity.

Cells of *B*. *pseudomallei* are able to adhere to host epithelial cells, commence invasion and induce apoptosis [40]. In this study, we used annexin V and PI staining to assay apoptosis and necrosis. We found that infection with strains H777 and C17 induced apoptosis of A549 cells more than did infection with strain M10. Infection with strain H777 induced the highest observed level of apoptosis by 12 hours p.i. (1.52%). Lengwehasatit et al. demonstrated the role of RpoS of *B*. *pseudomallei* in mitochondrial membrane-potential changes, caspase-3 activation and induction of apoptosis in infected mouse macrophages (RAW264.7) [41]. This information suggests an essential role for RpoS in the regulation of cell death in mouse macrophages. Additionally, a type III translocator protein, BipB was found to be involved in virulence of *B*. *pseudomallei*. Induction of apoptosis via the caspase pathway by *B*. *pseudomallei* required BipB [31]. In addition, Sun et al [42] demonstrated that *B*. *pseudomallei* induced caspase-1-dependent death in infected macrophages with the assistance of *bsa* Type III Secretion System (TTSS) leading to pore formation and cell death [42]. Nevertheless, a *B*. *pseudomallei* capsule mutant had a significantly greater ability to invade HeLa and A549 cells and had a greater ability to induce apoptosis in A549 cells than did wild type *B*. *pseudomallei* [30]. This data implies that the capsular polysaccharide of *B*. *pseudomallei* plays a minor role in induction of cytotoxicity or apoptosis in epithelial cells. The induction of apoptosis upon infection by pathogenic bacteria results from a complex interaction of bacterial proteins with cellular proteins, finally mediating apoptosis. Host cell apoptosis may aid the bacteria to attack the host and to gain access to the tissues. However, in some infections, apoptosis of mammalian cells significantly contributes to the host defense against bacteria, further indicating the role of apoptosis in host-pathogen interactions [43]. The current study is the first to provide direct evidence that *B*. *pseudomallei* biofilm motivated cell death by apoptosis relative to necrosis in human alveolar epithelial cells. Furthermore, the greater levels of apoptotic cells could be a result of the higher intracellular *B*. *pseudomallei* according to the higher adhesion and invasion capability of *B*. *pseudomallei* biofilm. The induction of apoptosis of the infected human alveolar epithelial cells may facilitate the pathogen in invading the surface barrier, allowing them to reach the submucosal layers. Moreover, apoptosis may also help the bacterium avoid phagocytosis, without the intense inflammatory response associated with host cell necrosis [44].

The pulmonary epithelium acts not only as a structural barrier against inhaled foreign organisms but also provides proinflammatory cytokines, chemokines to recruit immune cells including neutrophils and T cells to the site of infection [45, 46]. Our analysis suggested that A549 cells infected with biofilm wild type *B*. *pseudomallei* show a significantly higher level of IL-6 and IL-8 secretion than those infected with M10 (*p* < 0.05) (Fig 6). These results are in agreement with earlier studies [25, 47]. Moreover, increased levels of major pro-inflammatory cytokines IL-6, TNF-α and chemokine MCP-1, have been demonstrated from the murine lung epithelial cell line, primary lung epithelial cells and an inhalational murine infection model [24]. West and colleagues [48] suggested that murine pulmonary infection induced by inhalation of *B*. *pseudomallei* leads to increases in IFN-γ, IL-10, IL-1β, IL-6, KC and TNF-α in the lung 1 day after infection. Concentrations of proinflammatory cytokines TNF-α, IL-1, IL-6, and IL-12 were elevated in melioidosis patients [49, 50]. Our model mimics the clinical situation of acute melioidosis in that this disease can lead to induction of pro-inflammatory cytokines with high levels of cytokine secretion, leading to poorer prognosis for infected patients [50–52]. IL-8 is a potent neutrophil chemoattractant and activating factor produced by lung endothelial and epithelial cells and neutrophils have been implicated in the pathogenesis of many inflammatory lung diseases [53]. The correlation between biofilm phenotypes of *B*. *pseudomallei* and the elevation of IL-8 production of infected A549 cells extends our knowledge of biofilm in lung immunopathology.

In summary, our study highlights the relevance of biofilm in *B*. *pseudomallei* for interactions with the human lung epithelial cells. Successful adhesion, facilitated by biofilm, leads to higher rates of invasion and intracellular survival in these cells emphasized the role of biofilm in infected A549 alveolar epithelial cells. Moreover, the higher apoptotic cell death attribute to biofilm phenotypes of *B*. *pseudomallei* infection was also observed. Apoptosis of alveolar epithelial cells during *B*. *pseudomallei* infection may facilitate bacterial dissemination and reach to the host tissues [43]. In addition, a pulmonary *B*. *pseudomallei* infection can disseminate to distal organs with consequential development of bacteremia and poor prognosis [54, 55]. Furthermore, the *B*. *pseudomallei* biofilm provides a chance for this intracellular pathogen to trigger proinflammatory responses, particularly secretion of IL-6 and IL-8, from infected human lung epithelial cells. The elevation of these proinflammatory cytokines had various degrees of bronchopneumonia, with inflammation consisting of numerous neutrophils and a moderate number of macrophages in rhesus macaques and African green monkeys models that closely resembles that seen in acute human melioidosis [56]. These data suggested the vital role of biofilm in the intracellular pathogenesis of *B*. *pseudomallei*.
